# Aromatic L-Amino-Acid Decarboxylase Deficiency Screening by Analysis of 3-O-Methyldopa in Dried Blood Spots: Results of a Multicentric Study in Neurodevelopmental Disorders

**DOI:** 10.3390/genes14091828

**Published:** 2023-09-21

**Authors:** Susanna Rizzi, Carlotta Spagnoli, Melissa Bellini, Carlo Alberto Cesaroni, Elisabetta Spezia, Patrizia Bergonzini, Elisa Caramaschi, Luca Soliani, Emanuela Claudia Turco, Benedetta Piccolo, Laura Demuth, Duccio Maria Cordelli, Giacomo Biasucci, Daniele Frattini, Carlo Fusco

**Affiliations:** 1 Child Neurology and Psychiatry Unit, Department of Pediatrics, Presidio Ospedaliero Santa Maria Nuova, AUSL-IRCCS di Reggio Emilia, 42123 Reggio Emilia, Italy; susanna.rizzi@ausl.re.it (S.R.); carloalberto.cesaroni@ausl.re.it (C.A.C.); daniele.frattini@ausl.re.it (D.F.); carlo.fusco@ausl.re.it (C.F.); 2 Pediatrics and Neonatology Unit, Maternal and Child Health Department, Guglielmo da Saliceto Hospital, 29121 Piacenza, Italy; m.bellini@ausl.pc.it (M.B.); g.biasucci@ausl.pc.it (G.B.); 3 Pediatrics Unit, Department of Pediatrics, Azienda Ospedaliero-Universitaria Policlinico di Modena, 41125 Modena, Italy; elisabetta.spezia@aou.mo.it (E.S.); patrizia.bergonzini@aou.mo.it (P.B.); elisa.caramaschi@aou.mo.it (E.C.); 4 U.O.C. Neuropsichiatria dell’età Pediatrica, IRCCS Istituto Delle Scienze Neurologiche di Bologna, 40138 Bologna, Italy; luca.soliani2@unibo.it (L.S.); ducciomaria.cordelli@unibo.it (D.M.C.); 5 Child Neuropsychiatry Unit, Mother and Child Department, University-Hospital of Parma, 43126 Parma, Italy; eturco@ao.pr.it (E.C.T.); bpiccolo@ao.pr.it (B.P.); 6 R&D Biochemistry, Centogene GmbH, 18055 Rostock, Germany; laura.demuth@centogene.com; 7 Dipartimento di Scienze Mediche e Chirurgiche (DIMEC), Alma Mater Studiorum, Università di Bologna, 40138 Bologna, Italy

**Keywords:** AADC, Aromatic L-amino-acid decarboxylase deficiency, neurodevelopmental disorder, metabolic disease

## Abstract

Aromatic L-amino acid decarboxylase deficiency (AADCd) is a rare recessive metabolic disorder caused by pathogenic homozygous or compound heterozygous variants in the dopa decarboxylase (DDC) gene. Adeno-associated viral vector-mediated gene transfer of the human DDC gene injected into the putamen is available. The typical presentation is characterized by early-onset hypotonia, severe developmental delay, movement disorders, and dysautonomia. Recently, mild and even atypical phenotypes have been reported, increasing the diagnostic challenge. The aim of this multicentric study is to identify the prevalence of AADCd in a population of patients with phenotypic clusters characterized by neurodevelopmental disorders (developmental delay/intellectual disability, and/or autism) by 3-O-methyldopa (3-OMD) detection in dried blood spots (DBS). It is essential to identify AADCd promptly, especially within non-typical phenotypic clusters, because better results are obtained when therapy is quickly started in mild-moderate phenotypes. Between 2021 and 2023, 390 patients with non-specific phenotypes possibly associated with AADCd were tested; none resulted in a positive result. This result highlights that the population to be investigated for AADCd should have more defined clinical characteristics: association with common signs (hypotonia) and/or pathognomonic symptoms (oculogyric crisis and dysautonomia). It is necessary to continue to screen selected clusters for reaching diagnosis and improving long-term outcomes through treatment initiation. This underscores the role of newborn screening in identifying AADCd.

## 1. Introduction

Aromatic L-amino acid decarboxylase deficiency (AADCd, OMIM #608643), first described in 1990, is a rare inborn autosomal recessive metabolic disorder caused by pathogenic homozygous or compound heterozygous variants in the Dopa Decarboxylase (DDC) gene. The AADC enzyme catalyzes the final decarboxylation step in the synthesis of monoamine neurotransmitters. AADC enzymatic deficiency leads to a severe combined deficiency of serotonin and dopamine, and consequently of norepinephrine and epinephrine. 

Its global incidence is unknown exactly, but it is more prevalent in Asian populations (especially Taiwanese and Japanese), probably due to a founder effect [1]. The neonatal prevalence has been estimated at 1/42,000 [2], while a newborn screening program based on 3-O-Methyldopa (3-OMD) testing on dried blood spots disclosed a prevalence of about 1/32,000 in Taiwan [3]. In the high-risk population (i.e., patients with a suspicion for biogenic amine neurotransmitter disorder), a prevalence of approximately 0.112%, or roughly 1:900, was found [2], whereas in at-risk populations in Asia, the prevalence is 50% higher than in the non-Asian population [4]. In the European Union (EU), the estimated frequency of AADC is 1:116,000, corresponding to an estimated incidence of 45 newborns per year and to an estimate of 853 living patients in the EU [5]. Furthermore, population studies have recently been published that highlight the frequency of carriers of variants affecting the DDC gene using whole exome sequencing data from the Genome Aggregation Database (gnomAD). The worldwide frequency of heterozygous carriers of a pathogenetic or probably pathogenetic variant of the DDC gene is 0.17%; the highest frequency of carriers was reported, as already known, in East Asians at 0.78%, while the lowest was reported in the South American population at 0.07% [6]. It should also be considered that since it is an autosomal recessive disease, regardless of ethnic origin, the risk is increased in unions between blood relatives. This possibility has already been reported in scientific literature, where clinical and molecular findings of AADC deficiency in members of a non-Asian consanguineous pedigree are described. The estimated incidence of AADC deficiency was 1 in 1,374,129 worldwide and 1 in 65,266 in East Asians [7].

The phenotypic spectrum is broad, ranging from very severe to relatively mild phenotypes. Clinical manifestations of AADC deficiency include neurological and non-neurological symptoms (Table 1) [8]. The typical presentation is characterized by early-onset hypotonia, movement disorders (oculogyric crises, dystonia, and hypokinesia), developmental delay, and dysautonomia (nasal congestion, abnormal sweating, excessive drooling, and temperature instability). Pseudomyasthenic features, such as ptosis and fatigue with evening worsening, are frequent. Sleep disturbances, behavioral disorders (irritability, dysphoria, and autism-like symptoms), and gastrointestinal symptoms (gastroesophageal reflux, diarrhea, and constipation) are common. Hypoglycemic episodes in infancy may be associated, while epilepsy is rare. Moreover, mild and atypical phenotypes have been recently reported, increasing the diagnostic challenge. The great majority of described patients (about 80%) are classified as having a severe phenotype (no or very limited developmental milestones and being fully dependent on their caregiver) [9]. Likely, patients with mild or moderate phenotypes are at risk of being undiagnosed and misdiagnosed, a frequent issue in rare diseases. Our previous systematic review suggests that behavioral problems, such as autistic features, reported as less common by previous literature, are actually more represented than cardinal signs, such as ptosis or dystonia, but are not pathognomonic, further increasing the risk of underdiagnosis in AADCd patients [8].

The first laboratory diagnostic method is cerebrospinal fluid (CSF) neurotransmitter analysis. As expected, biochemical products decrease (low homovanillic acid and 5-hydroxyindoleacetic acid levels), while biochemical precursors increase (elevated L-dopa, 5-hydroxytryptophan, and 3-ortho-methyldopa levels). However, CSF analysis requires a lumbar puncture, which, being invasive, is not primarily considered for patients with unspecific presentations. A less invasive, faster, and cheaper test consists of measuring plasma 3-OMD levels in dried blood spots, which are usually high in patients with AADC deficiency. Finally, diagnosis can be genetically confirmed by detecting pathogenic and probably pathogenetic homozygous or compound heterozygous variants in the DDC gene [10].

As regards therapy, the pharmacological strategy historically used included selective dopamine agonists and monoamine oxidase inhibitors. Vitamin B6 (pyridoxine) is usually prescribed in this patient population as it represents an important cofactor of the AADC enzyme. It is also possible to use symptomatic treatments based on the signs and symptoms of the individual patient: anticholinergic agents for autonomic symptoms, melatonin for sleep disorders, and benzodiazepines for both sleep and movement disorders. Patients often require pharmacological polytherapy, which is sometimes able to relieve some symptoms but is not able to modify cognitive and motor performance or the overall prognosis.

Recently, gene therapy has become available. It consists of the administration, by a stereotaxic neurosurgical procedure, to the putamen or midbrain (substantia nigra), of an adeno-associated virus type 2 (AAV2), which contains the DDC gene (hAADC). The viral vector is internalized by endocytosis, then, through an endosomal-lysosomal degradation process, the viral capsid is broken up, and the viral genome with the DDC transgene is translocated to the nucleus. The DDC transgene persists as an extrachromosomal circular episome. The episome is transcribed, and therefore the AADC enzyme is produced. Gene therapy is a “una tantum” therapy administered only once in a lifetime. In July 2022, the European Medicines Agency (EMA) authorized PTC Therapeutics to market Eladocagene exuparvovec (Upstaza^®^) for intraputaminal administration, and in June 2023, the first Italian patient was treated at the Umberto I Hospital, Rome. Eladocagene exuparvovec is recommended in patients aged 18 months and older with a clinical, biochemical, and genetically confirmed diagnosis of AADC deficiency with a severe phenotype. Gene therapy, in addition to alleviating the disease-related signs and symptoms, leads to improved motor and cognitive performances and is generally well-tolerated [11]. In several studies, early diagnosis has been suggested to improve treatment efficacy, and individuals with mild and moderate phenotypes seem to show a better response [7,8]. Thus, in order for gene therapy to be an option and to modify the natural history of this disorder, prompt diagnosis is necessary, especially for mild-to-moderate and non-specific phenotypes.

The primary objective of this multicentric study is to identify, by 3-OMD level screening, the prevalence of mild/moderate, non-specific neurologic phenotypes potentially associated with AADC deficiency, especially within the phenotypic cluster characterized by neurodevelopmental disorders (developmental delay/intellectual disability and/or autism spectrum disorder). Getting diagnosed with a rare neurotransmitter disease can take a long time and therefore can prove very difficult for families and patients. Reducing diagnosis times must be a priority, especially for disorder in which gene therapy is available. A recent study highlighted that approximately half of patients were misdiagnosed before the diagnosis of AADC deficiency was reached. Notably, it was highlighted that approximately 80% of health care professionals took 12 months or more to refer subjects with AADC deficiency to an experienced specialist in their country, who then made the right diagnosis [12]. The rationale for investigating, through 3-OMD level’s screening, the prevalence of AADC deficiency in non-specific and therefore often common neurologic phenotypic clusters is the identification by a non-invasive, relatively fast, and cheap test of a disease that may present with non-specific and non-pathognomonic features and for which there is a gene therapy capable of modifying the prognosis.

## 2. Materials and Methods

All patients with non-specific neurologic phenotypes potentially associated with AADC deficiency, namely neurodevelopmental disorder (developmental delay/intellectual disability and/or autism spectrum disorder), who were referred to child neurology and pediatrics units of the Emilia Romagna region (Italy), have been collected between March 2021 and March 2023.

After obtaining parental consent, an additional blood spot for assaying 3-OMD levels analyzed by tandem mass spectrometry was picked up during scheduled routine blood tests. In the population examined in this multicentric study, no genetic validation was routinely carried out for the variants of the DDC gene. It is foreseen only for subjects who have obtained a positive screening result. Each subject enrolled in this multicentric study continued the diagnostic process on the basis of the choice of the attending physician.

### 2.1. Parental Consent

A consent form was signed for each subject participating in the 3-OMD screening. The parents of subjects undergoing 3OMD screening specifically consented to using their personal data for scientific research that focuses on the cause, early detection, and/or treatment of rare diseases in general. They also specifically consented to sharing their biochemical, genetic, and health data, including the results of the analysis, solely in de facto anonymized form, with external doctors, scientific institutions, and/or pharmaceutical companies for their own scientific research.

### 2.2. Biological Material

The material used was dried blood spot (DBS) filter cards prepared by dropping 50 µL of blood on CentoCard^®^ filter paper (Centogene GmbH, Cambridge, MA, USA); the spots were allowed to dry for 2–4 h at room temperature. The filter cards are the ideal material for shipping and long-term storage. Before analysis, 3.2 mm discs were cut from the homogeneous parts of the DBS using a DBS puncher (Perkin Elmer LAS GmbH, Rodgau, Germany). Each disc contains approximately 3.1 µL of blood.

### 2.3. Sample Preparation

For each subject, 5 DBS discs were cut and collected into a 96-deep-well plate (Thermo Fisher Scientific, Waltham, MA USA) by a pediatric nurse previously trained in the correct collection of the biological samples. Once dried, the samples are packaged and sent to the Centogene laboratory in Germany. The extraction solution consisting of aqueous DMSO solution and the internal standard solution (Lyso-Gb2 (Biotrend Chemikalien GmbH, Köln, Germany) in ethanol) were added to the plate. The plate was sonicated at room temperature, then incubated at 37 °C under agitation and again sonicated at room temperature. The samples were transferred to a PALL-8048 96-well filter plate with a PTFE membrane (WVR International GmbH, Darmstadt, Germany) on top of a 96-well V-shaped plate (WVR International GmbH). The cellular and paper debris were filtered by centrifugation. The V-shaped plate was covered with aluminum foil and inserted into the sample manager.

### 2.4. LC/MS Method

The samples were separated by liquid chromatography on a 4 µm AAA-MS column (EZ:faast, Phenomenex, Germany) using a Waters I-Class UPLC (Waters GmbH, Hessen, Germany). Solvents: FA in water (A) and FA in methanol/acetonitrile 1/1 (B). A linear gradient from 0 to 100% solvent B was used.

The UPLC was coupled with an AB-Sciex TQ-5500 (AB Sciex Germany GmbH mass spectrometer, Darmstadt, Germany). An MRM method was used for monitoring the analytes with the MRM transitions, 3-OMD (212.1 > 195 *m*/*z*), and Lyso-Gb2 internal standard (624.3 > 282.2 *m*/*z*).

### 2.5. Quantification

A 7-point calibration line was added to each plate before measurement. The preparation was similar to the samples, but the DBS discs were replaced with standard solutions of increasing 3-OMD (Sigma-Aldrich, Burlington, MA, USA) concentrations. The highest calibrator concentration and the upper limit of quantification amount to 13,000 nmol/L (blood). The cutoff was determined to be >1000 nmol/L 3-OMD blood concentration. This is the upper limit of the 95% confidence interval (modeled by the following equation: cut-off = mean + 2 × standard deviation) based on values from 110 control cases. An age-group-specific cut-off was not determined.

### 2.6. Method’s Validation

The assay was validated according to CAP/CLIA guidelines. Centogene has implemented a multi-discipline quality management system (QMS), which is recognized by Centogene CAP-laboratory, CAP ISO 15189 accreditation, and CLIA certification. During validation, samples of 110 control individuals (healthy or showing variants unrelated to AADC) and 80 genetically confirmed AADC cases have been measured. All 110 control cases showed a 3-OMD level below the cut-off, while all AADC-pathologic cases showed a 3-OMD level above the cut-off. This translates to a sensitivity and specificity of 100%.

### 2.7. Data’s Collection

A database was set up using Microsoft Excel 2016 to collect clinical and biochemical data. Each Child or Pediatric Neurology Unit involved in this multicentric study prepared its own database in the following way: the pediatric nurse who collected the biological sample entered the patient’s date of birth, sex, and the date of sending the biological sample into the format; subsequently, the study’s referring doctor entered the patient’s features and the result of the 3-OMD level’s screening into the clinical file. At the end of the study period, the databases of the Child Neurology Unit of Bologna, the Child Neurology Unit of Parma, the Pediatric Unit of Piacenza, and the Pediatric Unit of Modena were sent to the Child Neurology Unit of Reggio Emilia. Finally, the Child Neurology Unit of Reggio Emilia took care of extrapolating the anagraphic, clinical, and biochemical data.

All data were processed and reported in the database anonymously. General information about sex, ethnicity, and age was noted. Regarding the clinical features, we divided them into 6 classes of major neurological signs and symptoms (hypotonia, developmental delay/intellectual disability, autism spectrum disorder, epilepsy/EEG abnormalities, movement disorder, and dysautonomia) and catalogued them as present or absent. The database is available from the corresponding author upon reasonable request.

## 3. Results

A total of 245 patients were collected from the Reggio Emilia child neurology unit, 89 from the Piacenza pediatrics unit, 32 from the Modena pediatrics unit, 18 from the Bologna child neurology unit, and 6 from the Parma child neurology unit. A total of 390 patients adhered to the 3-OMD level’s screening: 273 males (70%) and 117 females (30%) aged between 2 months and 20 years of life. The mean age of the patients at the time of the screening was found to be 3.66, therefore equal to 3 years and 8 months. The median was in agreement with the average data, with a value of 3.58, equal to 3 years and 7 months. The patients were mostly Caucasian (306/390: 78%), followed by Arab (38/390: 10%) or Asian (29/390: 7%), while for the remainder it was not possible to identify the ethnicity with certainty (18/390: 5%). Regarding the clinical features, the results are reported in Table 2 and Table 3. Among the 37 patients with movement disorders, 7 have hypokinesia, 14 have dystonia, and 3 have both hypokinesia and dystonia. Only two patients presented oculogyric crises, one opsoclonus, and 5 did not further specify abnormal ocular movements. Of note, in our population there are 6 patients with a hyperkinetic movement disorder, a clinical feature not described in AADC deficiency, and therefore they were not included in the total movement disorder count. Among the 12 patients with dysautonomia, 5 have nasal congestion, 3 have excessive drooling, 3 have abnormal sweating, and 1 has temperature instability. Infantile episodes of hypoglycemia are reported in only two patients. None of the patients tested positive at the 3-OMD level’s screening.

## 4. Discussion

AADC deficiency is a disease that, due to its clinical characteristics and rarity, does not lend itself to prompt diagnosis. However, some of its typical symptoms are not pathognomonic, and therefore it can often be confused with other conditions that are decidedly more frequent and therefore probable, such as neuromuscular disorders, infantile cerebral palsies, and other neurodevelopmental disorders. Patients can remain undiagnosed for a long time, with a decrease in therapeutic efficacy and an inevitable worsening of the prognosis. The diagnosis of a rare disease is mandatory when disease-modifying therapy is available. It is known that, for AADC deficiency, better results are obtained when the treatment is promptly started in patients with mild to moderate phenotypes. Most of the subjects described until now present a severe and classic picture. Only a smaller proportion of patients described so far in the scientific literature present a mild-moderate phenotype, but they represent the group responding better to both pharmacological (selective dopamine agonists, monoamine oxidase inhibitors, vitamin B6) and gene therapy. The start of gene therapy determined a decisive turning point in the clinical history of this disorder, as it has proven effective in managing the signs and symptoms related to AADC deficiency while also improving both cognitive and motor performance. It is therefore essential to identify this disease early, especially within non-typical phenotypic clusters.

Given the scarce knowledge about the real prevalence of the disease with regard to mild-moderate phenotypes, the possibility of an atypical clinical presentation, and the availability of an effective and safe gene therapy, a screening project for AADC deficiency was started involving the Pediatric and Child Neurology Units of the Emilia Romagna Region (Italy). The aim of this multicentric study is to identify the prevalence of AADC deficiency in a phenotypic cluster characterized by non-specific neurologic phenotypes potentially associated with AADC deficiency, namely neurodevelopmental disorders (developmental delay/intellectual disability and/or autism spectrum disorder). A total of 390 patients have been screened from March 2021 until March 2023, and none tested positive at the 3-OMD level’s screening. The majority of our population are Caucasian male children with neurodevelopmental disorders (developmental delay/intellectual disability and/or autism spectrum disorder). Hypotonia, movement disorder, and dysautonomia, characteristic of AADC deficiency, are present in a minority of screened patients, respectively in 14%, 9%, and 2%. Epilepsy/EEG abnormalities are present in 27% of screened patients, which is higher than in AADC deficiency. Our multicenter study presents some limitations: First of all, the cohort studied is too small to be able to identify patients suffering from a very rare disorder such as AADC deficiency. Second, the criteria for the selection of subjects undergone 3-OMD level’s screening are broad. The result that no patients tested positive is consistent with the rarity of AADC deficiency and the estimated prevalence reported so far in the literature in the non-Asian population. However, our data allow us to conclude that the choice to screen a patient’s population for the most part presenting exclusively with a neurodevelopmental disorder is not an effective selection criterion. As a future prospect, a winning strategy could be to direct the screening for AADC deficiency to subjects with neurodevelopmental disorders associated with pathognomic symptoms such as dysautonomia or oculogyric crisis, present in more than 50% of patients with AADC deficiency. Another possibility to consider is screening a cohort of patients with neurodevelopmental disorders and hypotonia, a non-specific neurological sign that is typical of AADC deficiency. Since this is a rare condition, it is necessary to continue to screen as many patients with neurodevelopmental disorders associated with pathognomic symptoms such as dysautonomia or oculogyric crises as possible in order to understand the real prevalence within this phenotypic cluster and consequently to modify the clinical evolution with gene therapy’s administration. Another way to screen patients with a phenotype possibly associated with AADC deficiency is through whole exome sequencing (WES) [13]. Compared to the 3-OMD level’s screening, WES allows for greater diagnostic efficacy; however, it must be considered that almost all potentially identifiable neurodevelopmental disorders do not provide a treatment capable of modifying their evolution and prognosis. Furthermore, the execution of WES on a large scale of patients is certainly an effective but expensive method that is not easily accessible by all child neurology units, especially by pediatric units. Moreover, the time needed to obtain the results should not be underestimated. 3-OMD level’s screening can be obtained in a few days or at most weeks, while the WES requires a few months to be analyzed, studied, and concluded. Other studies are needed to truly evaluate the cost/benefit ratio of performing exome sequencing on a large population of subjects with signs and symptoms possibly associated with AADC deficiency in the short and medium term compared to the long term. Finally, regarding future perspectives, the best strategy is to identify AADC deficiency in the presymptomatic phase through the inclusion of this rare disorder in the national newborn screening. Prompt initiation of treatment before symptom onset would enhance the expected benefits and possibly obviate some signs and symptoms possibly associated with AADC deficiency. For this reason, more and more nations worldwide and, at the same time, more and more Italian regions are choosing to include the search for 3-OMD as a marker of AADC deficiency in newborn screening programs. It should be noted that, to date, there are no known cases of false negatives emerging from OMD level 3 screening; however, cases of false positives have been reported in the scientific literature. Therefore, all subjects who have obtained a positive result at the 3-OMD level’s screening will have to undergo genetic testing for validation [14].

## 5. Conclusions

AADC deficiency is a rare disease, but due to the therapeutic possibilities currently available, it should be investigated early in all children with signs and symptoms possibly associated with this condition, especially with early onset. A screening for AADC deficiency was performed on non-specific neurologic phenotypes potentially associated with AADC deficiency, namely neurodevelopmental disorders. To date, no patient enrolled in this Italian multicentric study has tested positive. The negative screening for AADC deficiency in examined subjects highlights that the population to be investigated for this rare condition should be a larger cohort and should have more defined clinical features, for example, the association with common signs such as hypotonia and/or pathognomonic symptoms such as oculogyric crisis and dysautonomia. Among these patients’ phenotypic clusters, it is necessary to continue to screen as many patients as possible with the aim of not missing diagnoses and improving long-term outcomes through prompt treatment initiation. Early diagnosis is deemed mandatory to achieve the greatest possible improvement with gene therapy, especially when a relatively fast, cheap, and easily accessible screening method is available, as in the case of the determination of plasma 3-OMD levels on blood-dried spots. This underscores the role of national newborn screening in identifying individuals with AADC deficiency. In the future, the most successful strategy will increasingly be to “play ahead” by identifying cases in a pre-symptomatic phase, thus being able to reduce the progression of AADC deficiency-related signs and symptoms and optimize the effectiveness of gene therapy. Early diagnosis is crucial for the patient and his family and allows access to target therapy.

## Figures and Tables

**Table 1:** Signs and symptoms reported in patients with AADC deficiency (estimated percentage for each clinical feature in the AADC deficiency population [10]).

Neurological	Non Neurological
Hypotonia (74%) Developmental delay (72%) Behavioral disorders (irritability, dysphoria, and autism-like symptoms) (30%) Movement disorders (oculogyric crisis (68%), dystonia (36%), and hypokinesia (42%)). Pseudo-myasthenic features (ptosis (26%) and fatigability) Epilepsy (4.5%) Sleep disorders (sleepiness or insomnia) (37%) Dysautonomia (nasal congestion, abnormal sweating, excessive drooling, hypotension, bradycardia, and temperature instability) (53%)	Infantile episodes of hypoglycemia (10%) Gastrointestinal symptoms (gastroesophageal reflux, diarrhea, and constipation) (19%)

**Table 2:** Clinical features in the screened population.

Clinical Features	Number of Patients with Clinical Features	Percentage of the Clinical Feature in Our Population
Hypotonia	54/390	14%
Developmental delay/Intellectual disability	260/390	67%
Autism spectrum disorder	158/390	41%
Epilepsy/EEG abnormalities	105/390	27%
Movement disorder	37/390	9%
Dysautonomia	12/390	3%

**Table 3:** Focus on data relating to the phenotypic cluster characterized by neurodevelopmental disorder.

Phenotypic Cluster	Number of Patients with Phenotypic Cluster	Percentage of the Phenotypic Cluster in Total Population
Neurodevelopmental Disorder	310/390	79%
Clinical subgroups of phenotypic cluster	Number of patients with clinical subgroups	Percentage of the clinical subgroups in phenotypic cluster
Developmental delay/Intellectual disability AND Autism spectrum disorder	108/310	35%
Developmental delay/Intellectual disability	153/310	49%
Autism spectrum disorder	49/310	16%

## Data Availability

Data are available upon request to the corresponding author.

## References

[1] Lee H.-F., Tsai C.-R., Chi C.-S., Chang T.-M., Lee H.-J. (2009). Aromatic l-amino acid decarboxylase deficiency in Taiwan. Eur. J. Paediatr. Neurol..

[2] Hyland K., Reott M. (2019). Prevalence of Aromatic l-Amino Acid Decarboxylase Deficiency in At-Risk Populations. Pediatr. Neurol..

[3] Chien Y.-H., Chen P.-W., Lee N.-C., Hsieh W.-S., Chiu P.-C., Hwu W.-L., Tsai F.-J., Lin S.-P., Chu S.-Y., Jong Y.-J. (2016). 3-O-methyldopa levels in newborns: Result of newborn screening for aromatic l-amino-acid decarboxylase deficiency. Mol. Genet. Metab..

[4] Lee H.-C.H., Lai C.-K., Yau K.-C.E., Siu T.-S., Mak C.M., Yuen Y.-P., Chan K.-Y., Tam S., Lam C.-W., Chan A.Y.-W. (2012). Non-invasive urinary screening for aromatic l-amino acid decarboxylase deficiency in high-prevalence areas: A pilot study. Clin. Chim. Acta.

[5] Hyland K., Peters M., Schu M., Erickson S., Croxford J., Whitehead N. Estimated prevalence of aromatic l-amino acid decarboxylase (AADC) deficiency in the United States, European Union, and Japan. Proceedings of the Annual Congress of the European Society for Gene and Cell Therapy.

[6] Park J.E., Lee T., Ha K., Cho E.H., Ki C.S. (2023). Carrier frequency and incidence of aromatic L-amino acid decarboxylase deficiency: A gnomAD-based study. Pediatr. Res..

[7] Graziano C., Wischmeijer A., Pippucci T., Fusco C., Diquigiovanni C., Nõukas M., Sauk M., Kurg A., Rivieri F., Blau N. (2015). Syndromic intellectual disability: A new phenotype caused by an aromatic amino acid decarboxylase gene (DDC) variant. Gene.

[8] Himmelreich N., Montioli R., Bertoldi M., Carducci C., Leuzzi V., Gemperle C., Berner T., Hyland K., Thöny B., Hoffmann G.F. (2019). Aromatic amino acid decarboxylase deficiency: Molecular and metabolic basis and therapeutic outlook. Mol. Genet. Metab..

[9] Wassenberg T., Molero-Luis M., Jeltsch K., Hoffmann G.F., Assmann B., Blau N., Garcia-Cazorla A., Artuch R., Pons R., Pearson T.S. (2017). Consensus guideline for the diagnosis and treatment of aromatic l-amino acid decarboxylase (AADC) deficiency. Orphanet J. Rare Dis..

[10] Rizzi S., Spagnoli C., Frattini D., Pisani F., Fusco C. (2022). Clinical Features in Aromatic L-Amino Acid Decarboxylase (AADC) Deficiency: A Systematic Review. Behav. Neurol..

[11] Kojima K., Nakajima T., Taga N., Miyauchi A., Kato M., Matsumoto A., Ikeda T., Nakamura K., Kubota T., Mizukami H. (2019). Gene therapy improves motor and mental function of aromatic l-amino acid decarboxylase deficiency. Brain.

[12] Badnjarevic I., Moyer K., Bertoldi M., Opladen T., Flint L. (2023). Navigating the Rare Neurotransmitter Disease Diagnosis: Insights from Patients and Health Care Professionals. J. Inherit. Metab. Dis..

[13] Riva A., Iacomino M., Piccardo C., Franceschetti L., Franchini R., Baroni A., Minetti C., Bisello G., Zara F., Scala M. (2023). Exome sequencing data screening to identify undiagnosed Aromatic l-amino acid decarboxylase deficiency in neurodevelopmental disorders. Biochem. Biophys. Res. Commun..

[14] Chen P.W., Hwu W.L., Lee N.C., Chien Y.H. (2023). Streamlined determination of 3-O-methyldopa in dried blood spots: Prospective screening for aromatic l-amino-acid decarboxylase deficiency. Mol. Genet. Metab..

